# The effect of the COVID-19 pandemic on the provision of outpatient clinic services in East Jerusalem hospitals: patients’ perspectives

**DOI:** 10.3389/fpubh.2023.1252449

**Published:** 2023-11-24

**Authors:** Muna Ahmead, Firas Daghlas

**Affiliations:** Faculty of Public Health, Al-Quds University, Jerusalem, Palestine

**Keywords:** accessibility, availability of resources, quality of care, attitudes, a patient preferences, East Jerusalem, outpatient clinics

## Abstract

**Background:**

Due to the decreased availability, accessibility, and quality of services, the COVID-19 pandemic has an impact on the healthcare system. In the wake of the COVID-19 pandemic, patients’ perceptions of healthcare have changed, and out-patient visits to clinics have decreased. As part of the COVID-19 outbreak in East Jerusalem, this study aims to assess how patients perceive the way that outpatient clinic services were delivered before and during COVID-19 outbreak.

**Methodology:**

Convenience sampling and self-reported questionnaires were used in a cross-sectional study. Three hundred people from three significant outpatient clinic hospitals in East Jerusalem-Al-Makassed Hospital, Augusta Victoria Hospital, and Sant-Joseph Hospital- made up the sample. Multivariate tests, frequencies, and percentages were used in the statistical analysis.

**Results:**

The results showed that the most of the participants (98.6%) had negative opinion when the current situation is compared with before the COVID-19 period in terms of accessibility, availability of resources, quality of care, attitudes and patient’s preference. Finally, multivariate analysis indicated a significant relationship between participant opinion and education level and participants with educational levels of 12 study years or less had more positive opinions of the delivery of the healthcare system during the COVID-19 outbreak period than the group with more than 12 study years. Also, the multivariate analysis revealed a significant `relationship between participant opinion and the duration of the illness as those with years of illness and less had more negative opinion toward the delivery of the healthcare system than those with more than 3 years of illness.

**Conclusion:**

This study found that when the current situation during the COVID-19 outbreak is compared to before the COVID-19 period in terms of accessibility, availability of resources, quality of care, attitudes, and patient preferences, the majority of the participants with chronic diseases or cancer had a negative opinion. Policymakers and health managers should enhance patient preferences and attitudes during the COVID-19 pandemic and other pandemics by increasing accessibility, availability of health resources, and the quality of healthcare.

## Introduction

The 2019 coronavirus illness (COVID-19) is a serious public health emergency (1). On March 11th, 2020, the World Health Organization (WHO) declared COVID-19 to be a “pandemic” due to its quick global spread. It is a highly contagious virus that can cause mild to severe symptoms, or even no symptoms at all, and it can be fatal to high-risk people like the older adults (2). It has numerous impacts on national politics, the socioeconomic system, and public health (3). Several nations losing control of the pandemic led to high death rates and insufficient medical care (2).

The “World Health Organization” (WHO) states that each national health system should be directed to accomplish three main objectives: being responsive to the population’s expectations, promoting good health, and fair financial contribution. The provision of equal access to high-quality services for both acute and chronic health issues, including services that successfully promote health and prevent disease as well as quick responses to emerging threats (such as the COVID-19 pandemic and the burden of non-communicable diseases), should be another requirement of every health care system (4). Actually, the hospital’s success is determined by its capacity to satisfactorily address the needs of its clients and earn their satisfaction (5). Therefore, providing high-quality care and ensuring patient safety will be very challenging during any pandemic (6).

In terms of quality, the COVID-19 pandemic, for instance, has a direct impact on the healthcare system and has led to a drop in the standard of care, a decrease in the number of people seeking treatment, and a shortage of supplies (7). Additionally, hospitals experience severe staffing shortages, shortages of medical resources like hospital beds, medications, and ventilators, as well as shortages of protective equipment (PPE), and medical supplies as a result of the pandemic’s sharp decline in equity and accessibility (1). For instance, during the pandemic’s peak, many countries had observed a significant decline in general practitioner appointments and specialist care attendance, which made it difficult to get access to physical and mental support, delayed the need for treatment, and resulted in a shortage of specialized care (8). Additionally, resources from routine care were diverted to handle the surge in new cases, and traditional quality and safety measures have gotten worse as a result of the rapidly evolving disease transmission patterns (9).

As a result of health systems giving COVID-19 patients priority, many regular, non-COVID-19 patients have not received adequate care and are dissatisfied with the services provided by the health system (10). According to a “pulse survey” conducted during the COVID-19 pandemic, 94% of the 135 nations reported disruptions in the provision of essential services between January and March 2021. These services included both mental health and non-communicable and communicable disease care (11). The failure to meet their medical needs led to several patients complaining. One study found that 1 in 5 individuals did not receive the required medical evaluation or care (12). Additionally, the number of visits from outpatients to the clinic has decreased throughout the COVID-19 lockdown period because outpatients would rather avoid exposure and the chance of contracting the coronavirus disease, which has an impact on routine screening, managing risk factors, and maintaining continuity of care for patients with chronic illnesses (13, 14). The Arsenault et al. study (15) found that during the COVID pandemic, outpatient visits overall decreased by 9–40% in 10 different countries, and visits for diabetes or hypertension decreased by over 20% in Chile, Haiti, Mexico, Nepal, South Africa, and Thailand (15). Outpatients who are at risk are reluctant to continue with their regular doctor visits, delaying or avoiding unnecessary visits because they are unable to make safe arrangements to continue their routine clinic consultations (16). Additionally, a study by Nez et al. (17) found that the availability of chronic treatment in these outpatient clinics decreased during the COVID-19 pandemic and chronic obstructive pulmonary disease (COPD), hypertension, and diabetes were the conditions most adversely impacted by the loss of access to healthcare (17). Moreover, a study done in Nigeria found that there was an increase in the percentage of people who had trouble accessing essential medicines, going from 10.6% before the COVID-19 lockdown to 35.2% during the lockdown, while 84.0% of the participants saw a worsening of their chronic health conditions as a result of the difficulty accessing essential medicines (18).

In Palestine, when the first cases were discovered in Palestine on March 5, 2020, the Palestinian Authority immediately proclaimed a State of Emergency and started effective national containment efforts to urge the populace to take precautions (19). During the COVID-19 pandemic, the healthcare system has been under a lot of stress. A severe lack of COVID-19 tests, sanitation and hygiene supplies, ventilators, and ICU beds revealed the weakness of the Palestinian healthcare system during the pandemic (20). The situation was also made worse by the deteriorating living conditions in the West Bank, Gaza Strip, and East Jerusalem, which included crowding, building restrictions, Israeli raids and arrests, home demolitions, and the lack of freedom of movement throughout Palestine (20). The population’s health and Palestinians’ ability to establish a cutting-edge healthcare system in East Jerusalem are in jeopardy due to this political unpredictability and socioeconomic instability (21). According to Israeli public health regulations, East Jerusalem is completely under Israel’s control and is isolated. Due to the effects of the ongoing occupation, illegal annexation, and systemic discrimination in the holy city, the situation in East Jerusalem deteriorated even before the start of COVID-19. In addition to lockdowns, closures, and limits, regulations were also implemented, which was important because patients were unable to access Israeli hospitals (21). So, the “East Jerusalem Hospitals Network” (EJHN), which consists of six Palestinian hospitals, is in charge of managing and caring for COVID-19 cases (20). Because of the ongoing underfunding and underdevelopment of Palestinian healthcare, Palestinian populations in East Jerusalem are especially vulnerable to the COVID-19 pandemic (22). Additionally, there are no COVID-19 testing facilities, and the information used to track the disease’s spread is false and unreliable (22).

Studies that evaluate the delivery of the healthcare system in outpatient clinics during the COVID-19 outbreak from the perspective of the patients are lacking in Palestine, especially in East Jerusalem. The purpose of this study is to assess the patient’s perception of the delivery of outpatient clinic services during the COVID-19 outbreak in East Jerusalem in terms of accessibility, resource availability, quality of care, attitudes, and patient preference. This study is essential because it has been determined that the primary administrative challenges in the healthcare setting are the inability to satisfy patient requests and the lack of patient cooperation in care decisions (23). The Palestinian Ministry of Health, decision-makers, and hospital administrators may find the findings of this study useful in planning for, containing, and responding to the COVID-19 emergency as well as future pandemics. According to our knowledge, this is the first study of its kind in Jerusalem.

## Materials and methods

### Study design

This study aimed to evaluate how patients felt about the delivery of services from outpatient clinics during the COVID19 outbreak in East Jerusalem hospitals. A cross-sectional design was used to accomplish the goal.

### Study settings and sampling

Patients (men and women) older than 18 years’ old who visited the outpatient clinics at three of East Jerusalem’s major hospitals were included in the study. The hospitals were Al-Makassed Hospital, Sant-Joseph Hospital, and Augusta Victoria Hospital. These medical facilities were selected because they provided care to the majority of coronavirus patients in East Jerusalem and had outpatient clinics for a range of chronic conditions, including cardiovascular diseases, rheumatoid arthritis, hypertension, diabetes mellitus, and cancer. The patents who could not read or write were excluded.

Four thousand five hundred and twenty five participants made up the target population of the current study. Computer software (PEPI-for-Windows) estimated the study sample for patients in each hospital using a proportional estimation method, and 355 participants were determined as the sample size^1^ according to the following criteria: 0.05 significance level, 95% confidence level, 50% response distribution, and 0.05 precision error. Three hundred and fifty-five participants were personally approached by the researchers in the outpatient clinics using a convenience sampling approach. The participants completed the questionnaire on their own. Data collection took place in 2020 from April to June.

### Data collection tool

A self-administered questionnaire was the tool used to collect the data and was developed by Ali Jadoo (2014) (24). The questionnaire consisted of three parts. There were three sections to the questionnaire. Age, gender, marital status, level of education, place of residence, income status, and occupation were among the socio-demographic factors in the first section. The patient’s medical history variables (clinic type, length of illness, frequency of patient visits per month, and COVID-19 infection) made up the second section.

The third part consisted of 17 items designed to assess patients’ opinions about the healthcare systems delivery during the COVID-19 outbreak and divided into 5 groups including accessibility (five questions), availability of resources (three questions), quality of care (four questions), patient’s attitude (three questions), and patients ‘preferences (two questions). A five-point Likert-type scale was used to score the closed comparative statements. Additionally, there were five different response options for each sentence (strongly agree, agree, unsure, disagree and strongly disagree), ranging from (1) “strongly agree” to (5) “strongly disagree”. Negative word questions were reverse scored (e.g., 1 = 5, 2 = 4, etc.) and these questions were (17, 18, 20, 29, 31).

On each of the scale’s overall dimensions, the respondents were split into two groups according to their opinions (positive and negative). As a result, dummy variables for (0) negative and (1) positive opinion were created and added from the 17 items’ original (1–5) (1–85) scores. On the basis of a median split (cut-off point), it was decided to categorize the summary score into two dependent variables: (0) for low or negative opinion, and (1) for high or positive opinion toward the delivery of health services before and during COVID-19 outbreak. The Cronbach’s Alpha reliability test for the overall scale was 0.70 which considered as acceptable. A committee of four public health experts reviewed the scale’s contents because it had not been previously tested in the Palestinian culture to make sure that the tool is culturally appropriate and no changes were done. The research team translated this study’s questionnaire first into Arabic, and then a certified medical translator translated it back into English. Before the survey was piloted with 10 patients to test for language clarity, both the original English questionnaire and the back translated version were examined by 4 experts to ensure that the translation was accurate.

### Data analysis

The data was analyzed by using the statistical package for Social Sciences (SPSS) version 23. The descriptive analysis including frequencies and percentages were calculated for socio-demographic and medical history related variables and for the questions whose answers were using the 5 point Likert scale. To find important contributing factors for people’s opinions in this study including sociodemographic variables and medical history variables, multivariable regression analyses were carried out.

### Ethical issues

The study current was conducted according to the Declaration of Helsinki principles. Ethical approval was obtained from the Ethical Committee at the School of Public Health/Al-Quds University (Ref No: 162/REC/2021). The participants were provided with the information sheet about the study including the aim of the study, objectives, and procedures. The participants informed that they had the right to refuse to participate in the study and their participation was anonymous. Also, the general directors of the selected three hospitals were formally approached by a letter that presented information about the proposed study and its purpose. Individual informed consent for participation in this study was obtained by their acceptance to fill in the questionnaire. Confidentiality and privacy were assured for all the participants.

## Results

In this study, 355 participants were personally approached to fill in the questionnaire and the response rate was 84.4%. Table 1 shows that among the participants, there were (65.3%, *n* = 196) women and (34.7%, *n* = 104) men. The average age of the participants (37.3%, *n* = 112) was between 18 and 40 years old, and the majority of participants (78.3%, *n* = 235) were married. Additionally, 53.1% of participants (*n* = 156) lived in urban areas, and 64.2% of participants (*n* = 192) finished their education in 12 years or less. Among participants, only (36.7%, *n* = 105) had a monthly income of $900 or more, while (36.4%, *n* = 104) had no income at all.

**Table 1:** Socio-demographic variables of the participants.

**#**	**Factors**		**Frequency**	**Percentage**
1	Hospital	Al-Makassed Islamic Charitable Association Hospital	89	29.7%
		Saint Joseph Hospital	97	32.3%
		Augusta Victoria Hospital	114	38%
2	Gender	Male	104	34.7%
		Female	196	65.3%
3	Age	18–40 years	112	37.3%
		41–50 years	88	29.3%
		More than 50 years	100	33.3%
4	Marital status	Single	40	13.3%
		Married	235	78.3%
		Other(divorce/widow)	25	8.3%
5	Educational level	12 study years or less	192	64.2%
		More than 12 study years	107	35.8%
6	Living place	City	156	53.1%
		Village	118	40.1%
		Camp	20	6.8%
7	Monthly income	No Income	104	36.4%
		Less 900$	77	26.9%
		900 $ and more	105	36.7%
8	Occupation	Employed	170	56.7%
		Unemployed	130	43.3%

According to Table 2, 24.8% of participants (*n* = 73) came from the diabetes clinic, 44.2% from the internal diseases clinic (*n* = 130), and 31% % from the cancer clinic (*n* = 91). When asked how long they had been ill, 35.8% of the participants (*n* = 100) said they had been sick for over 3 years. Only 67 subjects (24.4%, *n* = 67) were found to have a coronavirus infection, and regarding the frequency of clinic visits: (43.5%, *n* = 120) of patients went once a month.

**Table 2:** Medical history variables of the participants.

**#**			**Frequency**	**Percentage**
10	The clinic where you receive treatment is	Internal medicine clinic	130	44.2%
		Diabetes clinic	73	24.8%
		Cancer Clinic	91	31%
11	How long is your illness?	Less than 1 year	86	30.8%
		1–3 years	93	33.3%
		More than 3 years	100	35.8%
12	Number of visits to the clinic each month	Once per month	120	43.5%
		Twice and more per month	87	31.5%
		At least once every 2 months	22	8%
		At least once every 3 months and more	47	17%
13	Have you been infected with Corona virus	Yes	67	24.4%
		No	208	75.6%

**Figure 1:**
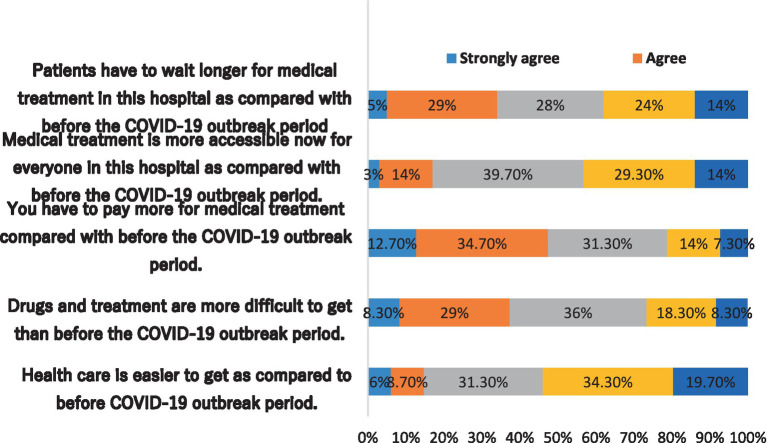
Accessibility to health care during COVID-19 outbreak.

Three of the five questions (questions 1, 4, and 5) were more likely to have’ negative opinions about access to healthcare during the COVID-19 pandemic (Figure 1). For instance, when it was stated that “Healthcare is easier to get as compared to before the COVID-19 outbreak period,” 54% of respondents (*n* = 162) disagreed and strongly disagreed.

**Figure 2:**
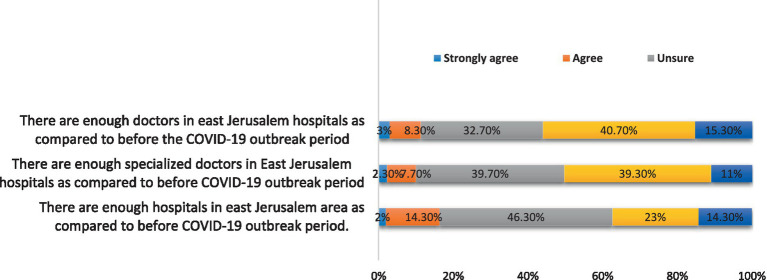
Availability of resources during COVID-19 outbreak.

The results also revealed that there were unfavorable answers to two of the three questions (1, 2) about the availability of resources during the COVID-19 outbreak. During the COVID-19 outbreak phase, in contrast to the period prior to it, 56% of the participants (*n* = 168) disagreed and strongly disagreed with the statement that “East Jerusalem hospitals had enough doctors” (Figure 2).

**Figure 3:**
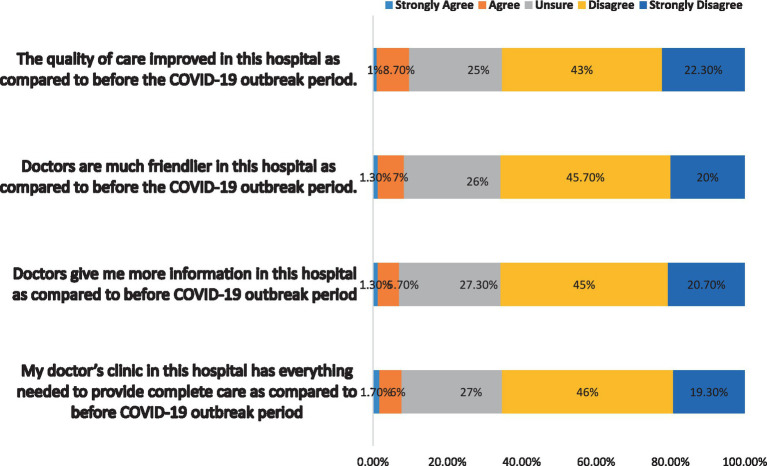
Quality of health care during COVID-19 outbreak.

Furthermore, the findings showed that all questions were more likely to have conveyed negative opinions about quality of health care delivered during the COVID-19 outbreak. For instance, 65.7% (*n* = 197) of participants disagreed and strongly disagreed with the statement “Doctors are much friendlier in this hospital as compared to before the COVID-19 outbreak period” and 65.3% (*n* = 196) of participants disagreed and strongly disagreed with the statement “The quality of care improved in this hospital as compared to before COVID-19 outbreak period” (Figure 3).

**Figure 4:**
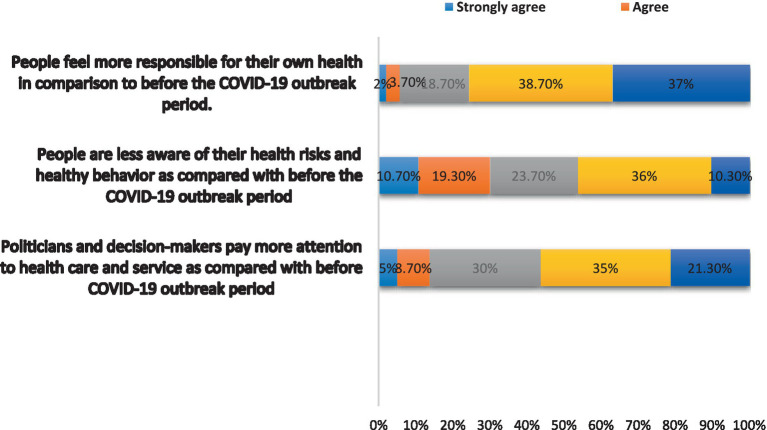
Attitudes during COVID-19 outbreak.

Moreover, the results were more likely to have showed a negative attitude toward each of the questions in this section. For example, the statement that “People feel more responsible for their health as compared to before COVID-19 outbreak phase” was strongly and strongly disagreed with by 75.7% of participants (*n* = 227). In response to the statement, “Politicians and decision-makers pay greater attention to health care and service as compared with before COVID-19 outbreak phase,” 56.3% of participants (*n* = 169) (disagreed and strongly disagreed) with this statement (Figure 4).

**Figure 5:**
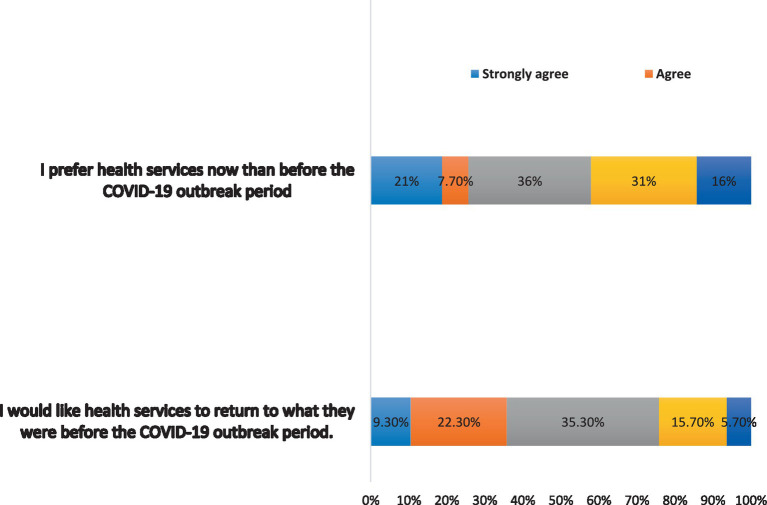
Preference during COVID-19 outbreak.

Finally, the findings were more likely to have showed that participants had a negative preference for the provision of health care during the COVID-19 outbreak, with 47% (*n* = 141) disagreeing and strongly disagreeing with the statement “I prefer health services now than before the COVID-19 outbreak period” (Figure 5).

### Respondents’ opinion by domains

Table 3 shows the overall respondents’ opinion by domains. Results were more likely to reveal that 98.6% of respondents more likely to have generally negative opinions about the delivery of health care services during the COVID-19 outbreak compared to the time before the COVID-19 in terms of accessibility, availability of resources, quality of care, preference and the attitudes.

**Table 3:** Frequency distribution of overall participant’s opinion by five domains.

**Domains**	**Mean**	**Standard deviation**	**Median**	**Positive opinion**		**Negative opinion**	
				**Frequency**	**Percentage**	**Frequency**	**Percentage**
Accessibility	15.6	2.84	15	29	9.66%	271	90.3%
Available resources	10.39	2.29	10	28	9.33%	272	90.6%
Quality	15.06	3.07	15	21	7%	279	93%
Attitude	10.80	1.77	11	79	26.3%	221	73.6%
Preference	6.74	1.25	6	17	5.66%	283	94.3%
Overall people view	58.61	7.14	58	4	1.3%	296	98.6%

Additionally, across all domains, the averages and percentages of (negative opinion) were highest. For example, 94.3% of participants were more likely to have a (negative opinion) about their preference for health care during the COVID-19 outbreak compared to the time before the COVID-19 outbreak, which is the first dimension. The second domain is healthcare quality, which is followed by attitude (73.6%), the availability of resources for healthcare (90.6%), and accessibility to healthcare (90.3%).

### Opinion by socio-demographic factor

According to multivariate analysis, there was only a significant relationship between participant opinion and education level (*p-*value 0.005). For instance, participants with educational levels of 12 study years or less were more likely to have more positive opinions of the delivery of the healthcare system during the COVID-19 outbreak period than the group with more than 12 study years ([OR] =0.536, 95% CI: 0.310–0.927, *p* = 0.005) (Table 4).

**Table 4:** Association between sociodemographic factors and respondent’s opinions.

**#**	**Factors**		**Positive opinion (%)**		**Negative opinion (%)**		**Value of *p***	**Wald**	**Sig**	**Exp(B)**	**95% C.I.**	
											**Lower**	**Upper**
			**Freq.**	**%**	**Freq.**	**%**						
1	Hospital	Al-Makassed Hospital	50	16.7%	39	13%	0.417	0.574	0.448	1.266	0.688	2.327
		Saint Joseph Hospital	46	15.3%	51	17%		0.181	0.671	0.879	0.486	1.591
		Augusta Victoria Hospital	57	19%	57	19%			Ref.			
2	Gender	Male	51	17%	53	17.7%	0.621	0.211	0.646	0.876	0.499	1.539
		Female	102	34%	94	31.3%			Ref.			
3	Age	18-40 Years	63	21%	49	16.3%	0.057	2.429	0.119	1.629	0.882	3.010
		41-50 Years	47	15.7%	41	13.7%		3.065	0.080	1.753	0.935	3.284
		Above 50 Years	43	14.3%	57	19%			Ref.			
4	Marital status	Single	19	6.3%	21	7%	0.50	1.430	0.232	0.514	0.172	1.531
		Married	120	40%	115	38.3%		0.797	0.372	0.669	0.276	1.617
		Other	14	4.7%	11	3.7%			Ref.			
5	Educational level	12 study years or less	86	28.8%	106	35.5%	**0.005**	4.986	0.026	0.536	0.310	0.927
		Above 12 study years	66	22.1%	41	13.7%			Ref.			
6	Living place	City	77	26.2%	79	26.9%	0.236	0.941	0.332	0.597	0.211	1.692
		Village	62	21.1%	56	19%		0.370	0.543	0.722	0.252	2.064
		Camp	13	4.4%	7	2.4%			Ref.			
7	Monthly income	No Income	55	19.2%	49	17.1%		1.929	0.165	1.599	0.825	3.100
		Less 3,000 NIS	39	13.6%	38	13.3%	0.834	0.138	0.710	1.131	0.592	2.161
		3,000 NIS and Above	54	18.9%	51	17.8%			Ref.			
8	Occupation	Employed	94	31.3%	76	25.3%	0.089	3.805	0.051	1.759	0.997	3.103
		Unemployed	59	19.7%	71	23.7%			Ref.			

*Multivariate logistic regression model: Adjusted for hospital, gender, age, marital status, educational level, living place, monthly income, and occupation. Significant *p-*values are in bold.

### Opinion by medical history factor

Furthermore, the multivariate analysis revealed only a significant `relationship between participant opinion and the duration of the illness. For instance, those with 1–3 years of illness and those with less than a year of illness during the COVID-19 outbreak period were more likely to have negative opinions more negative opinion toward the delivery of the healthcare system ([OR] = 0.545, 95% CI: 0.271–1.096, *p* = 0.044) and ([OR] = 0.505, 95% CI: 0.246-1.034, *p* = 0.044, respectively) than those with more than 3 years of illness (Table 5).

**Table 5:** Association between medical history factors and respondents’ opinion.

Variables						*P*-value	Wald	Sig	Exp(B)	95% C.I.for EXP(B)	
										Lower	Upper
		Positive opinion (%)		Negative opinion (%)							
#		Freq.	%	Freq.	%						
What type of disease do you suffer from?	Medical Diseases	59	20.1%	48	16.3%	0.511	0.010	0.994	1.004	0.327	3.088
	Cancer	47	16%	49	16.7%		0.042	0.837	0.701	0.024	20.664
	Diabetes mellitus	45	15.3%	46	15.6%			Ref.			
The clinic where you receive treatment is	Internal medicine clinic	73	24.8%	57	19.4%	0.959	0.045	0.832	0.702	0.027	18.585
	Diabetes mellitus clinic	34	11.6%	39	13.3%		0.247	0.619	0.412	0.012	13.606
	Cancer clinic	44	15%	47	16%			Ref			
How long is your illness?	Less than 1 year	40	14.3%	46	16.5%	**0.044**	3.490	0.006	0.505	0.246	1.034
	1-3 years	41	14.7%	52	18.6%		2.900	0.089	0.545	0.271	1.096
	Above 3 years	59	21.1%	41	14.7%			Ref			
Number of visits to the clinic each month	Once per month	67	24.3%	53	19.2%	0.393	1.737	0.187	1.759	0.760	4.073
	Twice and more visits per month	33	12%	54	19.6%		0.075	0.784	0.878	0.347	2.223
	At least once every 2 months	16	5.8%	6	2.2%		7.140	0.008	6.279	1.632	24.163
	At least once every 3 months and more	22	8%	25	9.1%			Ref.			
Have you been infected with Corona virus?	Yes	30	10.9%	37	13.5%	0.214	0.961	0.327	0.717	0.369	1.395
	No	109	39.6%	99	36%			Ref.			

Multivariate logistic regression model: Adjusted for: type of disease do you suffer from, the clinic where they receive treatment, duration of illness, number of visits to the clinic each month, and Have you been infected with Corona virus? Significant *p-*values are in bold.

## Discussion

In most developed and developing countries, evaluations of healthcare system from the viewpoints of the public or patients are rare (24). One of the most important tasks for healthcare organizations is to satisfy the needs and expectations of patients because doing so encourages patients to correctly and promptly follow doctor’s orders, which advances the process’ primary goal of accelerating the healing and recovery processes. In terms of accessibility, the availability of resources, the quality of care, attitudes, and people’s preferences, the results of the current study generally demonstrated that the majority of the participants had negative opinions when the current situation is contrasted to before the COVID-19 period (98.6%). These study’s findings were similar to those of a survey (25) which revealed that Americans had a negative opinion of the health system during COVID-19 and did not trust the public health system during the COVID-19 pandemic (26).

However, the results of this study disagreed with those of Grissom et al. study (2021), which found that COVID19 appeared to have had a positive effect on the overall level of patient satisfaction (26). Additionally, the results of the present study were at odds with those of a study by Bin Traiki et al. (2020), which was carried out in Saudi Arabia and found that patient satisfaction levels were high across all health domains, with generally positive surgical outcomes, demonstrating that all measures and policies put in place during the pandemic were beneficial for the patients (27). It is important to note that these two studies were carried out during the early stages of the COVID and the first 3–4 months of the pandemic, when the services were not significantly impacted by the COVID 19 pandemic and patients with non-COVID-19-related concerns avoided going to the hospital. The use of a different self-reported questionnaire and hospital method of administration may be the reason for the differences between our study and other studies. In addition, the level of satisfaction is also a subjective phenomenon that can vary greatly from patient to patient.

Additionally, the results of the current study are important because they provide further evidence of COVID-19’s negative impact on people with chronic conditions and cancer (13). In the current study, 90.3% of participants reported having a negative opinion about the access to healthcare services during the COVID-19 pandemic in comparison to the time before the pandemic. It was reported that the COVID-19 pandemic is associated with a variety of barriers to healthcare access and an increase in diabetic symptoms. The findings of the present study were also consistent with a study by Nez et al. (2021), which discovered that the difficulty in obtaining chronic treatments, such as those for COPD, diabetes, and hypertension, has worsened as a result of the decrease in healthcare access brought on by doctors’ “calls to duty” for urgent COVID-19 cases (17). For instance, 10 to 14% of people said their diabetic symptoms had gotten worse. Access issues were also connected to less frequent glucose monitoring frequency (13). Furthermore, those who have high blood pressure reported having more difficulty obtaining anti-hypertensive medication and treatment (20.3% in India, 8.6% in Hong Kong, and 6% in Korea). Another such group includes cancer patients, who are more likely than the general population to pass away from COVID-19-related severe sequelae, issues brought on by advanced age, and comorbidities (28, 29). Oncological patients with COVID-19 are predicted to have a mortality rate of 25.6% (30). According to data from Europe, more people with chronic conditions died at home as a result of not having access to life-saving medical treatments and the reallocation of healthcare funds to the control of COVID-19 (31, 32). The findings of the current study may indicate that people with chronic diseases should receive the appropriate services with high quality and without limited access or resources during the COVID 19 pandemic or any subsequent outbreak.

Additionally, determining the preferences of the patients may play a crucial role in providing successful planning, training, and caring programs (23). According to the current study’s findings, the preference for outpatient healthcare delivery during the COVID-19 outbreak was more likely to receive the highest percentages of (negative opinion) (94.3%). These findings were consistent with a study by Predmore et al. (2021), in which only 18.9% of participants chose the current health system delivery during COVID 19 outbreak such as using video visits and 61.7% preferred an in-person visit to the clinics (33). However, the results of the present study were in contrast to those of a study by Reicher et al. (2021), in which the participants were recruited through advertisements on websites intended for general social media users, the older adults, and individuals with chronic illnesses. The majority of study participants preferred the current delivery of health care services during the COVID-19 outbreak. For instance, (77%) agreed and strongly agreed that they would continue to use telemedicine services in the COVID-19 pandemic and would continue using these types of services in the future, and approximately 63% of participants were satisfied with the current health system’s delivery of telemedicine services (34). One possible explanation of the current study finding is that that the participants in this study might not have access to broadband internet and have limited digital literacy. Another factor is that the majority of participants had low incomes and educational backgrounds below those of four-year universities, which may have influenced their preference for outpatient healthcare delivery during the COVID-19 outbreak.

Another important prerequisite for the participation in the current study is the quality of care provided by healthcare systems, which has received more attention as a result of the pandemic. The quality of hospital procedures and services cannot be improved without actively pursuing patient satisfaction and closely attending to patients’ wants and expectations in terms of comfort as well as clinical treatments (23). The results of the current study revealed that 93% of participants had a negative opinion about the quality of care provided in outpatient clinics. This is consistent with a study by Tuczyska et al. (2022), who performed a systematic review of 12 studies (four from the United Kingdom and one each from Catalonia, Italy, Sweden, Poland, the Netherlands, France, Germany, and Belgium) to assess the quality of healthcare services in Europe during the COVID-19 pandemic. The study found that the COVID-19 outbreak adversely affected the quality of healthcare in the majority of European countries, with the exception of England. In England, the government’s actions had a positive impact on the quality of healthcare services, such as encouraging patients to register online or over the phone rather than in person. The plan was to respond to as many inquiries via phone or video call, but if a face-to-face meeting was required, it was scheduled for later that day. The use of home visits has also been successful in certain areas of England, particularly for patients who would find it difficult to travel. According to WHO (2020), improving the quality of health care during COVID-19 can minimize both direct and indirect mortality from outbreaks and illnesses that can be treated and avoided by vaccination. The provision of safe, efficient, and client-centered healthcare services was a problem for pandemic. Therefore, health systems should commit to identifying and attending to patients’ psychological, physical, and other needs over the course of their treatment.

Whether there is a healthy relationship between medical staff and patients is one of the key factors that affects the quality of care and, ultimately, the treatment’s outcome (35). Interestingly, the majority of patients did not feel satisfied with their interactions with their doctors, according to the results of the current study. The capacity of health care workers to develop enduring connections with patients that are defined by empathy has been proven to have a significant impact on patients’ recovery, perceived self-worth, distress, contentment, and hope (36). One of the most important aspects that affects the quality of therapy and, consequently, its efficacy is the degree to which patients and medical staff get along well (23). Furthermore, research has demonstrated that individuals with robust immune systems are those who receive caring empathy from their medical professionals (37). However, health care workers’ elevated levels of stress and worry during the COVID-19 outbreak, which impair their interactions with patients in addition to their fear of catching the corona virus, may be one explanation for this result (8, 38–40). These patients should therefore receive psychiatric care and assistance in outpatient clinics during any future pandemic.

According to a study by Okereket al. (2021), the safety of medical professionals working in the front lines of the COVID-19 pandemic is also a major concern, and a sharp decline in the availability of PPEs that are appropriate and scarce medical resources may make it challenging for people to access healthcare services (39). Our findings are consistent with those of a study by Nyasulu and Pandya (2020), which found that the COVID-19 pandemic had a direct impact on the health system during the pandemic that had a detrimental impact on its functionality and resource depletion in addition to diverting the health workforce, suspending services, reducing health-seeking behavior, and reducing the availability of supplies (41). According to Ahmed et al. study (2020), which compared healthcare access for those living and working in slum communities in Bangladesh, Kenya, Nigeria, and Pakistan before and during COVID-19, access to healthcare services, including preventive services such as immunization and reproductive, maternal and child health services, had decreased and been disrupted during the pandemic. Additionally, it was difficult for people to access healthcare facilities, because healthcare costs rose, and household income decreased (42).

Additionally, the results of the current survey also showed that most of the participants were more likely to have a negative attitude toward the delivery of healthcare during the COVID-19 outbreak. These findings were in contrast to a study conducted in India by Gopalakrishnan et al. (2021), which discovered that the majority of participants (84.2%) showed a positive attitude regarding COVID-19 prevention and that 93.0% of participants adhered to the advised safety procedures (43). The outcomes of the current study also did not agree with those of the study by Olum et al. (2020), which found that 74% of the participants had a positive attitude toward COVID-19 prevention (44). Additionally, according to Nguyen et al. (2021), the majority of participants (76.3%) had favorable attitudes, and more than half of the participants (57.7%) continued to practice good COVID-19 prevention (45). One possible explanation for the patients’ negative attitudes in the current study could be that the lack of medical services and supplies during the COVID-19 pandemic made it difficult for patients with chronic illnesses in East Jerusalem to feel secure and protected in the hospital environment. In a study conducted in Vietnam by Nguyen et al., it was found that participants who had learned enough information about the illness displayed positive attitudes and optimistic expectations for COVID-19 control. Hospitals also had to put in place a number of stringent pandemic control and prevention measures and policies, including the ban on visitors while a patient is in the facility, the need for a face mask and a medical declaration, the distribution of educational materials, hand hygiene kits, and social seclusion guidelines, as well as the suspension of inpatient visiting and the broadcasting of warnings inside hospital buildings. These actions made a significant contribution to the patients’ positivity and confidence regarding their general health condition as well as to their safety while they were in the hospital, which had a positive impact on the patients’ attitude (45).

Finally, multivariate analysis indicated a significant relationship between participant opinion and education level and participants with educational levels of 12 study years or less were more likely to have more positive opinions of the delivery of the healthcare system during the Covid-19 outbreak period than the group with more than 12 study years. It was found that patients expectations and perception of healthcare services are influenced by their level of education (46). Comparing literate and illiterate people, Biresaw et al.’s study (2021) found that literate people were 54% less likely to be satisfied with the service. This shows that patients who have relatively higher educational status have higher expectations because educated people are more critical of the services being provided and perceive some hospital activities as simple, they may not be as satisfied as less educated people (46). These findings were not similar to a study by Jadoo et al. (2014), which found a statistically significant relationship between lower education and negative opinions of the respondents. The results showed that those with less education were less likely to be satisfied with the healthcare system and more likely to express negative opinions (24). Jadoo et al. concluded that people with higher levels of education are less likely to incur out-of-pocket expenses due to being in good health and people with lower levels of education are more likely to do so due to being in poorer health (24).

Additionally, it is important to note that health literacy and education have frequently been linked (47–49). Health literacy focuses on practical skills (reading and math) or on the core competencies needed to gather and process health information (oral and written). Components of health literacy include data analysis, decision-making, reading and listening comprehension, and the ability to apply these skills in the right health situations (47). Health literacy would increase as educational levels did (50, 51). According to Kickbusch et al., people with less education frequently exhibit low health literacy (47). on other hand, highly educated individuals may also have poor health literacy abilities (48). People’s poor health literacy results in problems like insufficient use of preventative services, excessive diagnostic delay, insufficient adherence to medical advice, increased use of health services, increased risk of hospitalization, increased mortality rate, and significantly higher health-care costs (52, 53). In addition, people who have trouble understanding and utilizing health information and resources may have a difficult time managing chronic diseases (54). Poor cardiovascular health and a higher frequency of diabetic foot amputations, for example, have both been linked to low health literacy (55, 56) Jansen indicated that people with low levels of education might be able to avoid using health services if given the assistance to make informed choices (47).

Also, the multivariate analysis revealed a significant `relationship between participant opinion and the duration of the illness as those with 3 years of illness or less had more negative opinion toward the delivery of the healthcare system than those with more than 3 years of illness. Nikoloski et al. study reported that patients who received subpar care due to their health status or poor physical condition tend to be less satisfied with a variety of aspects of outpatient healthcare (47). This finding may be explained by the fact that patients who are diagnosed with chronic diseases for long time may have stable conditions than patients who are diagnosed for short period of time. Also, heavy users, such as those with chronic diseases or those in worse health, might interact with the healthcare system more frequently and have higher expectations for interactions with healthcare providers, which might have a negative impact on their satisfaction (47).

Also, the multivariate analysis revealed a significant `relationship between participant opinion and the duration of the illness as those with 3 years of illness or less had more negative opinion toward the delivery of the healthcare system than those with more than 3 years of illness (56). Nikoloski et al. study reported that patients who received subpar care due to their health status or poor physical condition tend to be less satisfied with a variety of aspects of outpatient healthcare (57). This finding may be explained by the fact that patients with chronic diseases who have been diagnosed for a longer period of time may have more stable conditions than patients who have only recently been diagnosed, which may have a positive impact on their satisfaction with the provision of healthcare services.

### Limitations

There are some limitations in this study. The sample included patients who attended outpatient clinics at the East Jerusalem hospitals, which may limit the generalization of the findings to other healthcare hospitals in Palestine. Furthermore, convenience sampling and cross-sectional design are barriers to making casual conclusions. Outbreak lockdowns decreased the number of patients attending to the outpatient clinics which decrease the number of participants in the current study. In addition, the provision of healthcare in East Jerusalem may have been inadequate even before the COVID pandemic and may have persisted throughout the pandemic, which may have had an impact on participants’ responses to the scale questions. As a result, qualitative research may be necessary to understand in more detail patients’ attitudes toward healthcare in Jerusalem during the COVID-19 pandemic.

Nevertheless, despite these limitations, providing our findings about the patient’s perspective of outpatient clinics services delivery during COVID-19 outbreak in East-Jerusalem represents a valuable contribution to the literature.

### Implications of the study

The health care management and system are likely to benefit practically from the study. Its conclusions can be applied to provide a set of suggestions that can be used to reduce the unfavorable effects of providing healthcare in outpatient clinics during public health emergencies like the COVID-19 pandemic in the Palestinian context. In times of emergency, it’s critical to support emergency preparedness capacity building to meet patient needs and expectations. In collaboration with the Ministry of Health and other healthcare providers, financial and technical support must be provided to the hospitals in East Jerusalem to improve accessibility, availability of resources, quality of care which may affect the attitudes and patient’s preference positively. People with cancer and chronic illnesses should also have access to high-quality, specialized medical care. Resources should be made available in a way that suits patients’ preferences to promote accessibility. Health policy makers should prioritize treating people with chronic conditions first in the event of a pandemic since these populations are currently at high risk for it, particularly patients with high levels of education and those whose illnesses have only lasted 3 years or less.

The results of the current study highlight how crucial the interaction between patients and medical professionals is. By emphasizing the individuality and needs expressed by patients, problematizing key elements that may contribute to care that is of high quality, safe, and patient-centered is made possible by understanding and discussing the patient’s perspective on healthcare. Future research may concentrate on how other approaches and cutting-edge tools, like the use of telehealth, video consultation, or online hospitals or outpatient’s clinics can be applied to any upcoming epidemic. Another crucial area to look into is the values and quality of these new services from the perspectives of patients and healthcare professionals for chronic diseases clinics compared to in-person visits.

## Conclusion

This study found that when the current situation during the COVID-19 outbreak is compared to before the COVID-19 period in terms of accessibility, availability of resources, quality of care, attitudes, and patient preferences, the majority of the participants with chronic diseases or cancer had a negative opinion. Policymakers and health managers should enhance patient preferences and attitudes during the COVID-19 pandemic and other pandemics by increasing accessibility, availability of health resources, and the quality of healthcare.

## Author’s note

MA is an assistant professor of mental health, and FD works as a staff nurse. FD has a master’s degree in health policy, and MA has a PhD in mental health. MA has experience conducting research on health services, PTSD, cancer, depression, fear of dying, quality of life, and other mental health issues and FD has experience conducting research on health organization, health policy and health services.

## Data availability statement

The original contributions presented in the study are included in the article/supplementary material, further inquiries can be directed to the corresponding author.

## Ethics statement

The studies involving humans were approved by Ethical Committee at the School of Public Health/Al-Quds University (Ref No: 162/REC/2021). The studies were conducted in accordance with the local legislation and institutional requirements. The participants provided their written informed consent to participate in this study.

## Author contributions

MA and FD designed the survey, developed the study tool, and participated in the study of advanced analysis. FD was responsible for data collection, data entry, and primary analysis. MA was responsible for writing the initial draft of the manuscript. All authors have read and agreed to the published version of the manuscript.

## Footnotes

^1^ www.raosoft.com
